# The interplay of intracellular calcium and zinc ions in response to electric field stimulation in primary rat cortical neurons *in vitro*

**DOI:** 10.3389/fncel.2023.1118335

**Published:** 2023-04-27

**Authors:** Abdullah J. Alshawaf, Sarah A. Alnassar, Futwan A. Al-Mohanna

**Affiliations:** ^1^Department of Physiological Sciences, College of Medicine, Alfaisal University, Riyadh, Saudi Arabia; ^2^Department of Cell Biology, King Faisal Specialist Hospital and Research Centre, Riyadh, Saudi Arabia

**Keywords:** electric field stimulation, calcium, zinc, membrane potential, cortical neurons

## Abstract

Recent pharmacological studies demonstrate a role for zinc (Zn^2+^) in shaping intracellular calcium (Ca^2+^) dynamics and vice versa in excitable cells including neurons and cardiomyocytes. Herein, we sought to examine the dynamic of intracellular release of Ca^2+^ and Zn^2+^ upon modifying excitability of primary rat cortical neurons using electric field stimulation (EFS) *in vitro*. We show that exposure to EFS with an intensity of 7.69 V/cm induces transient membrane hyperpolarization together with transient elevations in the cytosolic levels of Ca^2+^ and Zn^2+^ ions. The EFS-induced hyperpolarization was inhibited by prior treatment of cells with the K^+^ channel opener diazoxide. Chemical hyperpolarization had no apparent effect on either Ca^2+^ or Zn^2+^. The source of EFS-induced rise in Ca^2+^ and Zn^2+^ seemed to be intracellular, and that the dynamic inferred of an interplay between Ca^2+^ and Zn^2+^ ions, whereby the removal of extracellular Ca^2+^ augmented the release of intracellular Ca^2+^ and Zn^2+^ and caused a stronger and more sustained hyperpolarization. We demonstrate that Zn^2+^ is released from intracellular vesicles located in the soma, with major co-localizations in the lysosomes and endoplasmic reticulum. These studies further support the use of EFS as a tool to interrogate the kinetics of intracellular ions in response to changing membrane potential *in vitro*.

## Introduction

Calcium (Ca^2+^) and zinc (Zn^2+^) are important cations in neuronal and glial cells and are known to play structural and signaling roles to mediate diverse cellular functions ranging from cell division and differentiation to cell death (Berridge et al., 2000; Andreini et al., 2006; Foskett et al., 2007; Granzotto et al., 2020). The concentration of intracellular Ca^2+^ ([Ca^2+^]_*i*_) at rest is normally maintained around 100 nM and it is about 10,000-fold lower than that of extracellular environment, whereby any slight change in [Ca^2+^]_*i*_ would be perceived as specific signal coupled to a specific response (stimulus-response coupling). In addition, a multitude of molecular components are involved in Ca^2+^ signaling pathways, working in combinations to produce specific Ca^2+^ signals of different spatial and temporal profiles (Berridge et al., 2000). Similarly, the levels of labile Zn^2+^ are maintained considerably low in the cytosol with estimates corresponding to a range that extends to nanomolar ranges (Thambiayya et al., 2012; Granzotto et al., 2020). Unlike [Ca^2+^]_*i*_, a role for intracellular Zn^2+^ ([Zn^2+^]_*i*_) as a second messenger in certain cellular context was realized only recently (Yamasaki et al., 2007). On the other hand, the structural and catalytic functions of intracellular Zn^2+^ has long been realized (Thambiayya et al., 2012; Granzotto et al., 2020). This tight regulation of the intracellular concentrations of both ions is achieved via coordinated actions of various specialized group of proteins including channels, pumps, sensors, and buffers (McCall et al., 2000; Andreini et al., 2006; Thambiayya et al., 2012). In addition, much of the labile Ca^2+^ and Zn^2+^ is stored in the lumen of intracellular organelles such as the mitochondria, lysosomes, and endoplasmic reticulum (ER) or buffered by specialized proteins in the cytosol (Maret, 1994; Frederickson et al., 2000; Li et al., 2001; Outten and O’Halloran, 2001; Colvin et al., 2006; Hwang et al., 2008; Qin et al., 2011; Park et al., 2012; Khan et al., 2014; Lu et al., 2016). Recent studies indicate that Ca^2+^ and Zn^2+^ are intimately linked with each other, whereby Zn^2+^ contribute to shaping intracellular Ca^2+^ dynamics and vice versa specifically in excitable cells such as neurons and cardiac cells (Kiedrowski, 2012; Woodier et al., 2015; Sanford et al., 2019; Sanford and Palmer, 2020; Zhang et al., 2021).

Electric field stimulation (EFS) has been employed extensively *in vitro* to regulate neuronal behaviors such as excitability, migration, and regeneration (Alexander et al., 2006; Kobelt et al., 2014; Yan et al., 2014; Zhao et al., 2015; Hayashi et al., 2016; Pakhomov et al., 2017; Latchoumane et al., 2018; Tang-Schomer et al., 2018). It is well-established that EFS induces electrophysiological responses due to the invoked changes in the charged components of the membrane of the stimulated cells (Robinson, 1985; Tung and Borderies, 1992; Ye and Steiger, 2015). The electrophysiological responses may vary according to the cellular orientation and the inherent anisotropic orientation of the electric field (Tung and Borderies, 1992; Ye and Steiger, 2015). In addition, cationic currents at the plasmalemma of the stimulated cells largely depend on the activation and/or deactivation status of voltage gated ion channels (Robinson, 1985; Tung and Borderies, 1992), ultimately leading to intracellular interference with cation and/or anion homeostasis and other membrane bound receptor downstream signaling (Robinson, 1985; Taghian et al., 2015).

Several studies showed that exogenous EFS induces changes to the dynamic of [Ca^2+^]_*i*_, with varying findings regarding the kinetic and source of release (Alexander et al., 2006; Jahanshahi et al., 2014; Kobelt et al., 2014; Yan et al., 2014; Zhao et al., 2015; Hayashi et al., 2016; Pakhomov et al., 2017; Latchoumane et al., 2018; Tang-Schomer et al., 2018; Zhu et al., 2019; Meng et al., 2021). On the other hand, studies utilizing EFS to probe [Zn^2+^]_*i*_ are scarce, and only recently that pharmacological characterizations have been conducted in dissociated brain cultures *in vitro* to elucidate the kinetics of Zn^2+^ signaling in neurons and the consequential downstream effects (Hwang et al., 2008; Kiedrowski, 2012; Sanford et al., 2019; Sanford and Palmer, 2020). Accordingly, we investigated whether changing the excitability of the membrane by EFS can alter the dynamics of [Ca^2+^]i and [Zn^2+^]i signaling and whether an interplay between the two ions can be uncovered. Our data revealed that EFS induced hyperpolarization of the membrane which was concurrently accompanied by a rise in [Ca^2+^]_*i*_ and [Zn^2+^]_*i*_. The EFS-induced rise in Ca^2+^ and Zn^2+^ seemed to be due mainly to release from intracellular stores, and that the dynamic profile is indicative of an interplay between the two ions. These studies are important toward building an understanding of the kinetic of Ca^2+^ and Zn^2+^ signaling in response to changing excitability of neurons by EFS *in vitro*.

## Materials and methods

### Preparation of primary cortical cultures

The rat brain tissue dissociation protocols were approved by the Animal Care and Use Committee of the King Faisal Specialist Hospital and Research Centre (RAC#: 2190006). Primary cortical cultures were obtained from brains of 6–8 weeks rats (white, Wistar Rat). Cortices were isolated and enzymatically digested in HBSS media (Ca^2+^ and Mg^2+^ free, Thermo Fisher Scientific, Waltham, MA, United States) containing 2 mg/ml Papain, followed by adding 1:1 HBSS media and centrifugation. The palleted tissue was resuspended in HBSS media, dissociated mechanically by pipetting and passed through 70 μM strainer, centrifuged and plated onto Poly-d-lysine coated coverslips in Neurobasal media (Thermo Fisher Scientific, Waltham, MA, United States) supplemented with 2% B27 (Life Technologies, South San Francisco, CA, United States), 1% GlutaMax (Thermo Fisher Scientific, Waltham, MA, United States), 200 μM Glucose solution (Thermo Fisher Scientific, Waltham, MA, United States) and 1% Pen/strep (Thermo Fisher Scientific, Waltham, MA, United States). Cultures were maintained at 37°C and 5% CO_2_. Typically, approximately 2.7 × 10^6^ cells were seeded on 25 mm glass coverslips in 35 mm cell culture dishes. Medium was changed after 24 h to remove unattached cells. Experiments were conducted after 5 days post plating of the cortical cultures in order to avoid cultures being taken over by glial cells.

### Immunofluorescence

Cells were fixed in 3.7% Formaldehyde for 20 min at 4°C and then washed briefly in PBS. Cultures were permeabilized with 0.1% Triton-X- 100 in PBS (PBT) for 5 min and then blocked in 5% BSA in PBT (block buffer) for 60 min at room temperature. Samples were then incubated with primary antibody (diluted in the block buffer) overnight at 4°C. The following primary antibodies were used: mouse anti-MAP2ab (cat# MA5-12823, Thermo Fisher Scientific, Waltham, MA, United States), and rabbit anti-GFAP (cat# ab7260, Abcam, Cambridge, United Kingdom). Following three 5 min washes in PBT, secondary antibodies (1:1000 diluted in the block buffer) were applied for 1 h at room temperature. All samples were counterstained with 49,6-diamidino-2-phenylindole (DAPI; 1 μg/ml, Sigma Aldrich, Burlington, MA, United States). Samples were then mounted onto glass slides with 5 μl of moviol aqueous mountant followed by viewing and image capturing under Zeiss florescence microscope using ZEN imaging software.

### Electric field stimulation

**FIGURE 1:**
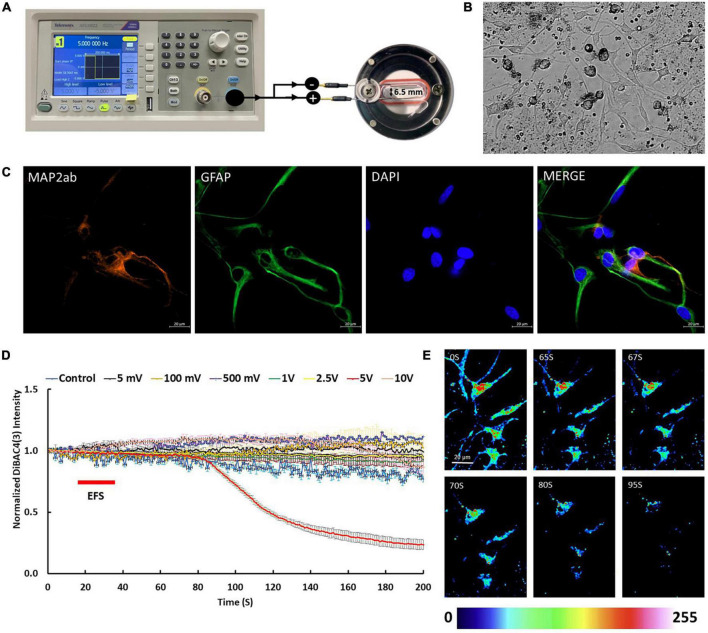
Electric field stimulation (EFS) protocol and cell cultures used in this study. **(A)** The experimental set up for delivery of EFS to the rat cortical cultures. Cells on coverslips were transferred into EFS chamber (on the right) which has two electrodes, separated by distance of 6.5 mm. Each electrode has two ends, one end is immersed in the media (700 μl) and the other end extends outside of the chamber. To apply EFS, the electrodes extending outside of the chamber were connected to a function generator (on the left). The function generator delivered rectangular pulse waves with a width of 200 ms at frequency of 5 Hz. **(B)** A phase contrast image of rat cortical cultures 5 days post plating. **(C)** Cortical cultures showed expression of neuronal marker MAP2ab (red) and glial marker GFAP (green). Scale bar = 20 μm. **(D)** The graph shows average intensity changes of the membrane potential dye DiBAC4(3) in response to EFS with different voltages, namel ±5 mV, ±100 mV, ±500 mV, ±1 V, ±2.5 V, ±5 V, and ±10 V. The EFS was applied at second 20 of the experiment for a duration of 20 s. Experiments were conducted in three independent brain preparations, each with three technical repeats. Data shown in **(D)** represent the average of one independent experiment and is expressed as normalized fluorescence intensity ratio relative to the averaged three images obtained prior to EFS. **(E)** Sequential confocal images of DiBAC4(3) labeled cortical neurons at the indicated time intervals corresponding to before, during and after exposure to EFS with ±5 V (calculated EFS intensity of 7.69 V/cm). Scale bar = 20 μm. Intensity changes in **(E)** are color-coded whereby high levels are red and low levels are blue.

Primary cultures on coverslips were transferred into an EFS chamber (Warner Instrument, Holliston, MA, United States). The chamber has two electrodes, separated by distance of 6.5 mm; each electrode has two ends, one end is immersed in the media and the other end extends outside of chamber (Figure 1A). The experimental chamber volume was 700 μl. To apply EFS, the electrodes were connected to a function generator (Tektronix, Beaverton, OR, United States) (Figure 1A). The function generator delivered ±5 mV to ±10 V, biphasic rectangular pulse wave that has a duration of 200 ms with a frequency of 5 Hz (Figure 1A) which produced different electric field potentials. EFS was applied for a total duration of 20 s *in situ* while recording a time lapse using Zeiss confocal imaging microscopy to monitor changes in the intensities of Ca^2+^ indicator Fluo-4 acetoxymethyl (AM) ester (Fluo-4 AM, cat# F14201, lot# 1524820, Thermo Fisher Scientific, Waltham, MA, United States), Zn^2+^ indicator, Fluozin-1 acetoxymethyl (AM) ester (Fluozin-1 AM, cat# F24181, MW 701.59, lot# 165983848, Thermo Fisher Scientific, Waltham, MA, United States), or membrane potential sensitive dye, bis-(1,3-dibutylbarbituric acid)-trimethine oxonol [DiBAC4(3), cat# B438, MW 516.64, Thermo Fisher Scientific, Waltham, MA, United States]. The EFS parameters used in this study did not cause cell death as evaluated using trypan blue exclusion test, propidium iodide staining and absence of membrane blebbing.

### Live cell microscopy

Cells were loaded with either Ca^2+^ indicator Fluo-4 AM (1 μM, Thermo Fisher Scientific, Waltham, MA, United States), Zn^2+^ indicator Fluozin-1 AM (1 μM, Thermo Fisher Scientific, Waltham, MA, United States) or membrane potential sensitive dye DiBAC4(3) (5 μM, Thermo Fisher Scientific, Waltham, MA, United States) in freshly prepared regular Krebs-HEPES buffer (pH 7.4) for 30 min at 37°C as previously described (Al-Saif et al., 2011; Shamseldin et al., 2017; Al-Mutairy et al., 2019). Krebs-HEPES buffer contained 120 mM NaCl, 1.3 mM CaCl_2_, 1.2 mM MgSO_4_, 4.8 mM KCl, 1.2 mM KH_2_PO_4_, 25 mM HEPES, and 0.1% BSA. All salts, HEPES and BSA were prepared from respective powders dissolved in Ultrapure (deionized-distilled) water (Resistivity is 15 MΩ.cm at room temp) produced by the Milli-Q^®^ Integral Water Purification Systems using M Quantum TIX filter (cat# QTUMOT1 × 1, Merck KGaA, Germany). The system removes all trace ions including Zn^2+^). For imaging of organelles, cells were loaded in regular Krebs-HEPES buffer with ER-Tracker Blue-White DPX (1 μM, 20 min at 37°C, Thermo Fisher Scientific, Waltham, MA, United States), LysoTracker Red DND-99 (1 μM, 10 min at 37°C, Thermo Fisher Scientific, Waltham, MA, United States), or MitoTracker Red CMXRos (1 μM, 1 min *in situ*, Thermo Fisher Scientific, Waltham, MA, United States). For experiments with the zinc chelator Tetrakis-(2-pyridylmethyl)ethylenediamine (TPEN), cells were loaded in regular Krebs-HEPES buffer containing 50 μM TPEN. For experiments in Ca^2+^ free medium, cells were loaded with specific indicator in Ca^2+^ free Krebs-HEPES buffer in which the addition of CaCl_2_ was replaced with supplementation of 1 mM ethylene glycol-bis(β-aminoethyl ether)-N,N,N’,N’-tetraacetic acid (EGTA). Image acquisition and analyses was performed using CLSM software (Zeiss META 510) or ZEN imaging software under Zeiss spinning wheel confocal microscope. Serial images were acquired at approximately 1 s intervals. A baseline recording was first taken then the stimulus was delivered at second 20, whereby cells were either stimulated with 25 mM KCl, 10 μM diazoxide or EFS as described in EFS method section for duration of 20 s. Imaging of cells exposed to EFS was performed in room temperature. For analysis, we performed background subtraction then we calculated ratio changes by normalizing intensity changes to the averaged intensity of the first three images before delivering the stimulus. Although our cultures contained many other cell types including glia, we targeted neurons only. This was made easier by using high resolution confocal fluorescence imaging which allowed us to identify neurons from other cells present in the isolated cultures.

### Statistical analysis

Paired samples *t*-test was used for baseline comparative data and two-tailed student *t*-test was used for inter-group comparisons. A *p* ≤ *0.05* was considered significant.

## Results

### Electric field stimulation alters the membrane potential

**FIGURE 2:**
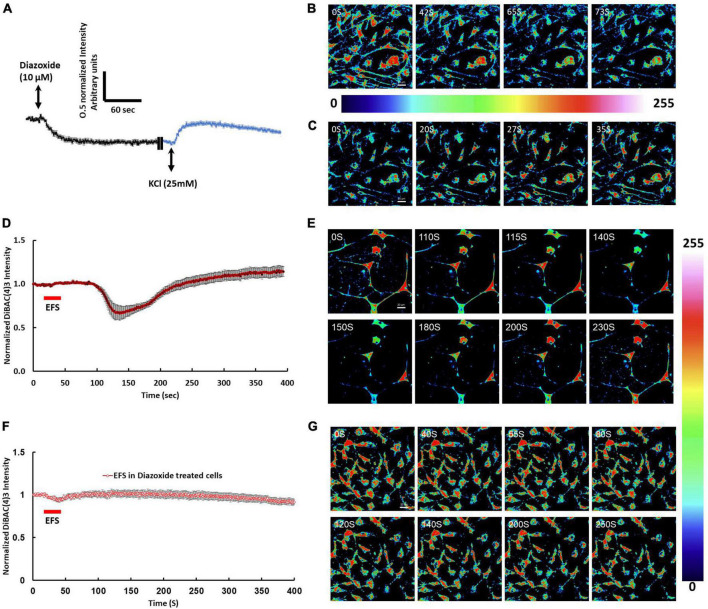
Membrane potential changes in response to pharmacological stimulation and EFS. **(A)** The graph shows average intensity changes of the membrane potential dye DiBAC4(3) in response to pharmacological stimulation to induce hyperpolarization or depolarization of the membrane using Diazoxide or KCl, respectively in rat cortical cultures. The graph shows traces of two sequential experiments where, in the first experiment, cells were stimulated with 10 μM Diazoxide followed by washing in Krebs buffer then in the second experiment, cells on the same field of view were stimulated with 25 mM KCl. Diazoxide and KCl were added at second 20 of the time lapse recording experiment. Data shown in **(A)** represent the average of one independent experiment and is expressed as normalized fluorescence intensity ratio relative to the averaged three images obtained prior to EFS. **(B)** Sequential confocal images of DiBAC4(3) labeled cortical neurons at the indicated time intervals corresponding to before, during and after addition of 10 μM Diazoxide. **(C)** Sequential confocal images of DiBAC4(3) labeled cortical neurons at the indicated time intervals corresponding to before, during and after addition of 25 mM KCl. Scale bar = 20 μm. Intensity changes in **(B,C)** are color-coded whereby high levels are red and low levels are blue. **(D)** The graph shows average intensity changes of DiBAC4(3) in response to EFS with intensity of 7.69 V/cm. The EFS was applied at second 20 of the experiment for a duration of 20 s. Data shown in **(D)** represent the average of one independent experiment and is expressed as normalized fluorescence intensity ratio relative to the averaged three images obtained prior to EFS. **(E)** Sequential confocal images of DiBAC4(3) labeled cortical neurons at the indicated time intervals corresponding to before, during and after exposure to EFS with ±5 V. Scale bar = 20 μm. **(F)** The graph shows average intensity changes of DiBAC4(3) in response to EFS with ±5 V in cells pretreated with 10 μM Diazoxide for 10 min. The EFS was applied at second 20 of the experiment for a duration of 20 s. Data shown in **(F)** represent the average of one independent experiment and is expressed as normalized fluorescence intensity ratio relative to the averaged three images obtained prior to EFS. **(G)** Sequential confocal images of DiBAC4(3) labeled cortical neurons at the indicated time intervals corresponding to before, during and after exposure to EFS with ±5 V. Scale bar = 20 μm. Intensity changes in **(E,G)** are color-coded whereby high levels are red and low levels are blue. Experiments were conducted in three independent brain preparations, each with three technical repeats. EFS, electric field stimulation.

Immunofluorescence of isolated cortical cell cultures from rat brains revealed a mixture of cell types expressing MAP2ab and GFAP consistent with the anticipated mixed population of neuronal and glial cells (Figures 1B, C). DiBAC4(3) loaded cultures were stimulated with different voltages including ±5 mV, ±100 mV, ±500 mV, ±1 V, ±2.5 V, ±5 V, and ±10 V at frequency of 5 Hz for 20 s starting at second 20 of the time lapse recording. EFS of cells loaded with DiBAC4(3) exhibited voltage dependent fluorescence changes consistent with changes in membrane potential (Figures 1D, E). Results showed that there were no significant changes in the intensity of DiBAC4(3) in cells stimulated ±5 mV, ±100 mV, ±500 mV, ±1 V, ±2.5 V, and ±10 V compared to unstimulated control (Figure 1D). A significant reduction in the intensity of DiBAC4(3) was observed in cultures stimulated with ±5 V, suggesting that an EFS with an intensity of 7.69 V/cm induces hyperpolarization rather than depolarization of the membrane (Figures 1D, E). In order to ascertain that the decrease in fluorescence intensity was indeed due to hyperpolarization, we challenged our cells with the ATP-sensitive potassium channel (K_ATP_) activator diazoxide [a hyperpolarizing agent that opens potassium channels and allows potassium efflux in a variety of cells (Langheinrich and Daut, 1997)]. We found that treatment of cells with diazoxide (10 μM) caused a significant decrease in DiBAC4(3) fluorescence which is consistent with hyperpolarization. Challenging the same cells with 25 mM KCl (after washing with fresh Krebs medium) invoked a clear increase in fluorescence intensity which is consistent with depolarization (Figures 2A–C). We next investigated the effect of the K_ATP_ activator on the EFS induced hyperpolarization. We found that prior treatment of cortical culture cells with diazoxide (10 μM) abolished the EFS-induced hyperpolarization (Figures 2D–G). The transient nature of the EFS-induced hyperpolarization is clearly demonstrated in Figure 2D. It is noteworthy that almost all of the cells tested exhibited EFS-induced hyperpolarization.

### Electric field stimulation induces a rise in intracellular calcium

**FIGURE 3:**
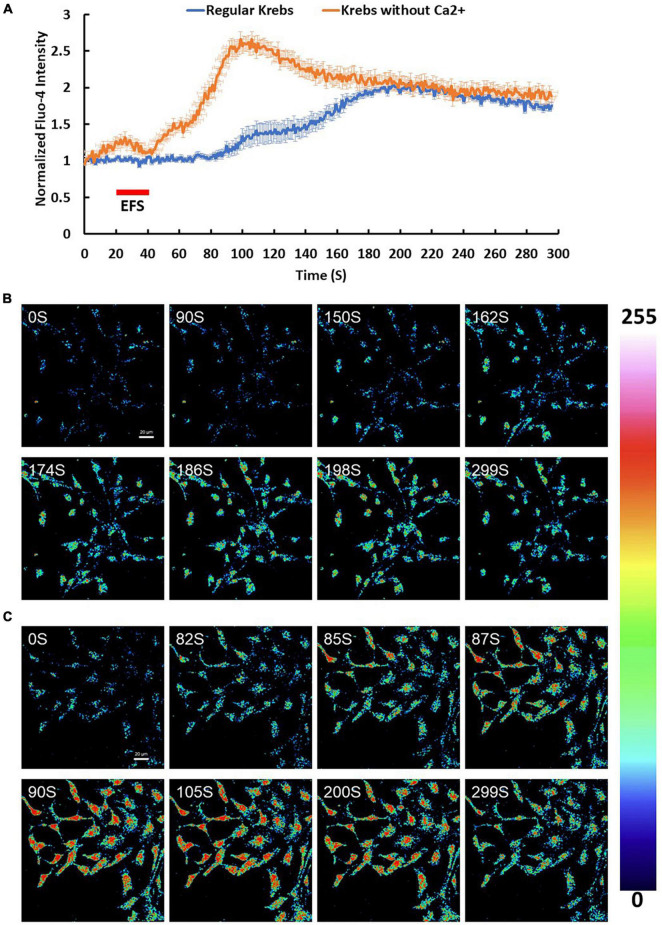
Calcium (Ca^2+^) changes in response to EFS. **(A)** The graph shows average intensity changes of Ca^2+^ indicator Fluo-4 AM in response to EFS in regular Krebs buffer (blue) and in Krebs buffer depleted from Ca^2+^ (red). The EFS was applied at second 20 of the experiment for a duration of 20 s. Experiments were conducted in three independent brain preparations, each with three technical repeats. Data shown in **(A)** represents the average of three independent experiments and is expressed as normalized fluorescence intensity ratio relative to the averaged three images obtained prior to the addition of stimulus. **(B,C)** Sequential confocal images of Fluo-4 AM labeled cortical cells at the indicated time intervals corresponding to before, during and after exposure to EFS with intensity of 7.69 V/cm **(B)** in regular Krebs buffer and **(C)** in Krebs depleted from Ca^2+^. Scale bar = 20 μm. Intensity changes in **(B,C)** are color-coded whereby high levels are red and low levels are blue. EFS, electric field stimulation.

To investigate changes in [Ca^2+^]_i_, cortical culture cells were loaded with Ca^2+^ indicator Fluo-4 AM and imaged by time lapse confocal microscopy. Fluo-4 AM loaded culture cells then were stimulated with an electric field of 7.69 V/cm at frequency of 5 Hz for 20 s (Figures 3A, B). Cortical cultures exhibited a heterogeneous Ca^2+^ response. 33% of the cells doubled their intracellular Ca^2+^ levels reaching a maximum intensity of 2.22 ± 0.04 in response to EFS (*p*-value ≤ 0.001, *n* = 3). The response was slow with a lag time between the EFS and the Ca^2+^ rise (end of EFS stimulation to the initial Ca^2+^ rise) being 70.90 ± 5.19 s with the intracellular Ca^2+^ slowly decaying to the minimum intensity at an average rate of 0.36% ± 0.024 per second (Figures 3A, B). The contribution of extracellular and intracellular calcium pools to the EFS-induced Ca^2+^ rise was investigated by performing parallel experiments in the absence of extracellular Ca^2+^ by bathing the cells in Ca^2+^ free Krebs-HEPES buffer containing 1 mM EGTA. Under such conditions, around 90% of cells doubled their intracellular Ca^2+^ levels in response to EFS with an average maximum intensity of 3.12 ± 0.09 (*p*-value ≤ 0.001, *n* = 3) (Figures 3A, C). The response was relatively faster with an average lag time between the EFS and the initial Ca^2+^ rise corresponding to 27.45 ± 1.71 s with the intracellular Ca^2+^ decaying to stimulatory levels at the rate of 0.79% ± 0.066 per second (Figures 3A, C and Supplementary Figure 4). These results suggest that the source of EFS-induced Ca^2+^ rise is most likely intracellular rather than from extracellular sources. In contrast, depolarization with extracellular KCl (25 mM) evoked an instant change in the intensity of Fluo-4 AM with a maximum corresponding to around 1-fold increase, suggesting Ca^2+^ influx upon activation of voltage gated calcium channels following depolarization of neurons (*n* = 3, data is not shown).

### Electric field stimulation induces a rise in intracellular zinc

**FIGURE 4:**
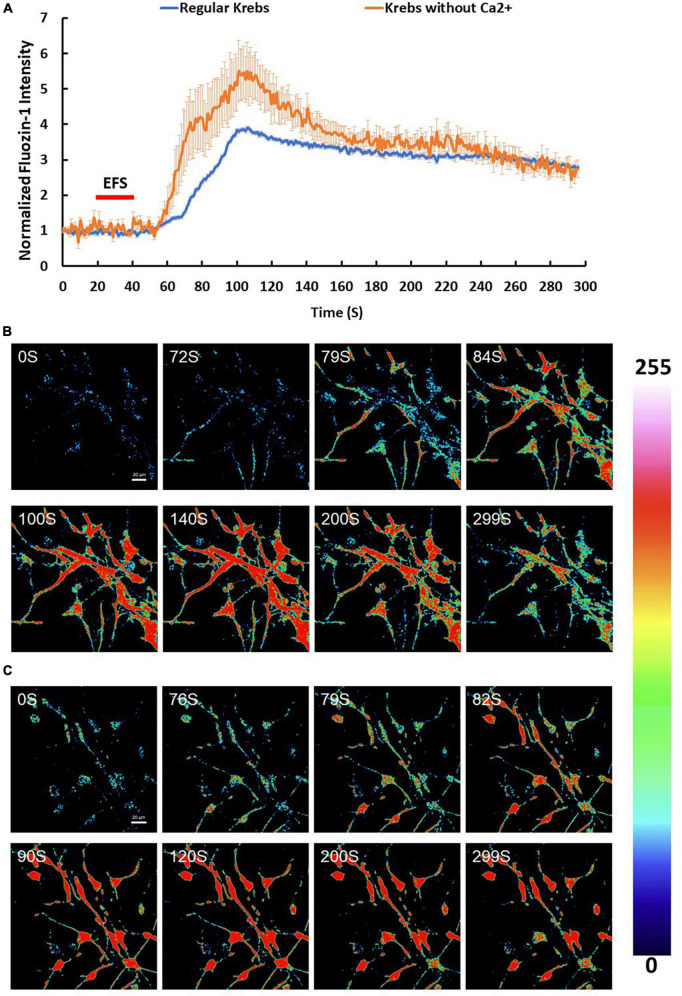
Zinc (Zn^2+^) changes in response to electric field stimulation (EFS). **(A)** Average intensity changes of Zn^2+^ indicator Fluozin-1 AM in response to EFS in regular Krebs buffer (blue) and in Krebs buffer depleted from Ca^2+^ (red). The EFS was applied at second 20 of the experiment for a duration of 20 s. Experiments were conducted in three independent brain preparations, each with three technical repeats. Data shown in **(A)** represent the average of three independent experiments and is expressed as normalized fluorescence intensity ratio relative to the averaged three images obtained prior to the addition of stimulus. **(B,C)** Sequential confocal images of Fluozin-1 AM labeled cortical cells at the indicated time intervals corresponding to before, during and after exposure to EFS with intensity of 7.69 V/cm **(B)** in regular Krebs buffer and **(C)** in Krebs depleted from Ca^2+^. Scale bar = 20 μm. Intensity changes in **(B,C)** are color-coded whereby high levels are red and low levels are blue.

**FIGURE 5:**
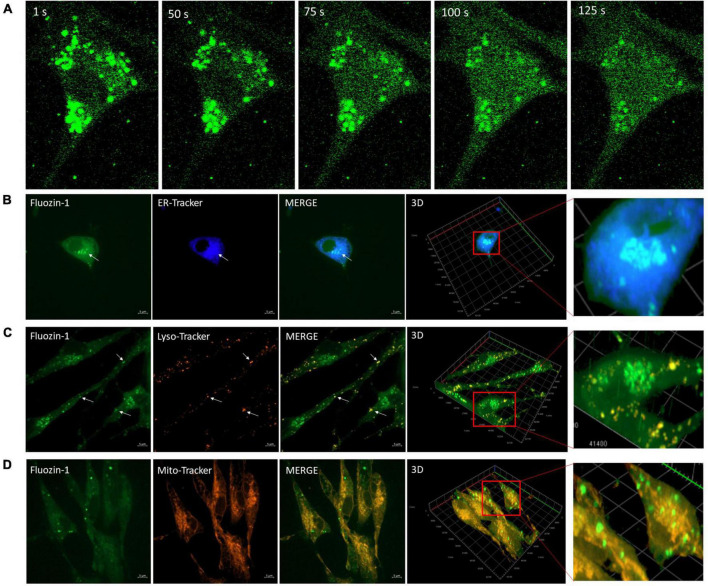
Vesicular bodies release zinc (Zn^2+^) in response to electric field stimulation (EFS). **(A)** Sequential images (100 × magnification) of Fluozin-1 AM labeled cortical cells at the indicated time intervals corresponding to before, during and after exposure to EFS with intensity of 7.69 V/cm. EFS was applied at second 20 of the experiment for a duration of 20 s. **(B)** Microscopic images of live cortical cells co-loaded with Fluozin-1 AM and ER-Tracker Blue-White DPX to identify co-localization of Zn^2+^ enriched vesicles (green) with the ER (blue). An example for co-localization is indicated by the white arrow. Scale bar = 5 μm. **(C)** Microscopic image of live cortical cells co-loaded with Fluozin-1 AM, and LysoTracker Red DND-99 to identify co-localization of Zn^2+^ enriched vesicles (green) with lysosomes (red). Examples for co-localizations are indicated by white arrows. Scale bar = 5 μm. **(D)** Microscopic image of live cortical cells co-loaded with Fluozin-1AM, and MitoTracker Red to identify co-localization of Zn^2+^ enriched vesicles (green) with mitochondria (red). Scale bar = 5 μm.

Since Zn^2+^ is intimately linked to Ca^2+^ homeostasis in many cell types, we investigated the effect of EFS on intracellular zinc using the Zn^2+^ indicator Fluozin-1 AM. We found that 96% of cells showed an average maximum increase of Fluozin-1 intensity of 4.94 ± 0.18 relative to baseline (*p*-value ≤ 0.001, *n* = 5) (Figures 4A–C). The EFS-induced Zn^2+^was inhibited by treatment of cells with the Zn^2+^ chelator TPEN prior to EFS stimulation (Supplementary Figure 1). Whether this EFS-induced rise in [Zn^2+^]_i_ was dependent on extracellular Ca^2+^ was investigated by applying EFS protocol to Fluozin-1 AM loaded cells in Ca^2+^ free-Krebs-HEPES buffer containing 1 mM EGTA. Under such conditions nearly all the cells within the randomly chosen fields of view responded to EFS with an average maximum of Fluozin-1 intensity corresponding to 8.25 ± 0.88 relative to baseline (*p*-value ≤ 0.001, *n* = 4) (Figures 4A–C). Further analyses revealed a significant statistical difference between the Fluozin-1 average maximum responses in the presence of extracellular Ca^2+^ compared with its absence, whereby the average maximum intensity was higher under extracellular Ca^2+^ free conditions (*p*-value = 0.002). Stimulation of Fluozin-1 AM loaded cells with 25 mM KCl, on the other hand, had no apparent effect on the intensity of the Fluozin-1 AM (*n* = 3, data is not shown), suggesting no release of Zn^2+^ in response to depolarization of the membrane. Given that the medium bathing the cells, Krebs-HEPES buffer, did not contain Zn^2+^, the source of the EFS-induced rise in Zn^2+^ observed in our experiments must be intracellular. In a series of experiments using high resolution imaging of Fluozin-1 AM loaded cells, we identified vesicular cytoplasmic organelles in our isolated cortical neurons displaying high intensity of Fluozin-1 AM fluorescence (Figure 5A). This apparent accumulation of Fluozin-1 AM in membrane bound cytoplasmic organelles dissipated after EFS stimulation with concomitant increase of fluorescence in the cytoplasm surrounding the organelles (Figure 5A). These Zn^2+^ containing vesicles showed major co-localizations with the ER, and lysosomes and minimal co-localizations with the mitochondria (Figures 5B–D).

### The interplay between EFS-induced hyperpolarization, calcium transients and zinc transients

Since the absence of extracellular calcium caused a significantly higher EFS-induced intracellular Ca^2+^ and intracellular Zn^2+^ transients we investigated the effect of EFS on membrane hyperpolarization in the absence of extracellular Ca^2+^. In a series of experiments, we found that EFS invoked faster and stronger hyperpolarization in calcium free Krebs-HEPES containing 1 mM EGTA than normal Krebs-HEPES medium (Supplementary Figure 2). In contrast to the transient hyperpolarization observed in normal Krebs-HEPES medium, the hyperpolarization observed under calcium free Krebs-HEPES medium was not transient. The possibility existed that the membrane hyperpolarization was responsible for the changes in Ca^2+^ and Zn^2+^. We used the chemical hyperpolarizer diazoxide to test this possibility. We found that diazoxide had no apparent effect on intracellular Ca^2+^ and Zn^2+^ (Supplementary Figures 3A–C). This data suggests that hyperpolarization *per se* was not responsible for the changes in Ca^2+^ and Zn^2+^.

## Discussion

In this study, we investigated the dynamic of [Ca^2+^]_i_ and [Zn^2+^]_i_ in response to altering excitability of the membrane using EFS in dissociated rat cortical cultures. Our results demonstrate that an EFS with an intensity of 7.69 V/cm induces membrane hyperpolarization with concomitant transient rise in [Ca^2+^]_i_ and in [Zn^2+^]_i_. Our findings suggest that: (i) the source of this EFS-induced Ca^2+^ and Zn^2+^ rise is intracellular, and (ii) the dynamic of the release may involve an interplay between the two ions. Evidence for the latter is drawn from the finding that removal of extracellular Ca^2+^ significantly augmented the release of intracellular Ca^2+^ and Zn^2+^. Further characterizations of the source of Zn^2+^ identified membrane bound vesicular organelles with high Fluozin-1 intensity. These organelles were found to be localized in the cell body and exhibited dispersed co-localization with the ER, and lysosomes.

Electric field stimulation -induced alteration to the [Ca^2+^]_i_ and [Zn^2+^]_i_ is unlikely to be due to the effect of transmembrane potential which itself is dependent on multiple factors including cellular orientation, direction and orientation of the electric field and activation or deactivation of voltage gated ion channels (Ye and Steiger, 2015). It is also well-understood that the EFS interferes with charged components of the membrane including membrane bound receptors and ionic distribution and ultimately affects cation and/or anion homeostasis (Robinson, 1985; Taghian et al., 2015; Ye and Steiger, 2015). It has been shown that the membrane exhibits high resistance to the EFS and, as a result, induces clustering of membrane proteins and receptors, leading to subsequent activation of various transmembrane signaling pathways (Cho et al., 1999; Leppik et al., 2020). The fact that chemical hyperpolarization had no apparent effect on [Ca^2+^]_i_ and [Zn^2+^]_i_ suggests that hyperpolarization *per se* was not responsible for the [Ca^2+^]_i_ and [Zn^2+^]_I_ transient reported in this study. Our finding that cortical culture cells exhibited stronger and more sustained hyperpolarization in the absence of extracellular calcium may suggest a link between extracellular calcium and hyperpolarization. However, while changes in calcium can influence the activity of ion channels and contribute to changes in the membrane potential, they are not typically the primary cause of hyperpolarization. Rather, they are often involved in downstream signaling pathways that are triggered by changes in the membrane potential.

Many studies employed EFS *in vitro* to investigate the long-term effects on neuronal behavior and function for therapeutic purposes (Alexander et al., 2006; Jahanshahi et al., 2014; Kobelt et al., 2014; Yan et al., 2014; Zhao et al., 2015; Hayashi et al., 2016; Pakhomov et al., 2017; Latchoumane et al., 2018; Tang-Schomer et al., 2018; Zhu et al., 2019; Meng et al., 2021). Several of these studies examined the role of Ca^2+^ signaling in mediating some of the observed cellular responses induced by EFS (Alexander et al., 2006; Jahanshahi et al., 2014; Kobelt et al., 2014; Yan et al., 2014; Zhao et al., 2015; Hayashi et al., 2016; Pakhomov et al., 2017; Latchoumane et al., 2018; Tang-Schomer et al., 2018; Zhu et al., 2019; Meng et al., 2021). An EFS with an intensity of 5 V and frequency of 10 Hz for a duration of 30 min have been shown to induce a rise in [Ca^2+^]_i_ and consequently promote neurite outgrowth in cultured dorsal root ganglia neurons (Yan et al., 2014). Further pharmacological characterizations suggested that the source of this EFS-induced [Ca^2+^]_i_ rise was both intracellular release from internal stores and extracellular through voltage gated Ca^2+^ channels (Yan et al., 2014). In another study, EFS of high intensity (20–25 V/cm) and high frequency was found to induce a rise in [Ca^2+^]_i_ in cultured hippocampal neurons (Riquelme et al., 2011). Consequently, this rise in [Ca^2+^]_i_ was found to induce generation of hydrogen peroxide and stimulate activity of NF_–*k*_B, a transcription factor known for its important role in dendritic arborization and plasticity (Riquelme et al., 2011). Further characterizations revealed that the source of this [Ca^2+^]_i_ rise was intracellular via activation of ryanodine receptors (Riquelme et al., 2011). These studies showed an instant increase in [Ca^2+^]_i_ following application of EFS. Whilst we acknowledge that pharmacological characterizations are lacking in our study, the kinetic of Ca^2+^ release reported by Riquelme et al. (2011) was different from our study. Our data indicated that only one third of the cells, under “regular” conditions, responded to EFS with a Ca^2+^ rise and that the rise was slow with a considerable lag between the EFS and the Ca^2+^ rise. This may be attributed to differences in EFS parameters applied, differences in cell types or different functional context.

Our study showed a rise in [Zn^2+^]i in response to EFS in cortical neurons and that the source of the release was intracellular stores. The cytosolic sources of labile Zn^2+^ in neurons can be either Zn^2+^ liberated from proteins such as metallothioneins or Zn^2+^ released from various intracellular organelles and compartments (Frederickson et al., 2000; Sensi et al., 2003; Colvin et al., 2006; Hwang et al., 2008; Qin et al., 2011; Park et al., 2012; Lu et al., 2016). Our studies indicate that Zn^2+^ enriched vesicles localized within the soma of cortical cells contributed to EFS-induced rise in [Zn^2+^]_i_. Two possibilities exist; (i) the high fluorescence intensity of Fluozin-1 in the vesicles reflects high Zn^2+^ content and/or (ii) the high intensity reflects different intra-vesicular environment that changes the spectral characteristics of the dye independently of Zn^2+^. The latter would seem unlikely since Fluozin-1 AM intensity is highest in pH 7 and decrease at more acidic or basic pH (Martins et al., 2020). Our co-localization experiments showed that most of the zinc enriched vesicles co-localized with the ER, and lysosomes while less co-localizations have been observed in the mitochondria. This is consistent with previous studies showing that Zn^2+^ enriched vesicles in cultured neurons and astrocytes are mainly enriched in secretory vesicles, endosomes, and lysosomes (Sensi et al., 2003; Colvin et al., 2006; Hwang et al., 2008; Lu et al., 2016). Other intracellular sources may also have contributed to the liberated Zn^2+^ such as metallothioneins, but this warrants further investigations.

Accumulating evidence highlights the important roles for Zn^2+^ and Ca^2+^ interactive signaling in several functional contexts that extend from modulating cellular excitability to mediating cellular injury and cell death (Claiborne et al., 1989; Frederickson and Danscher, 1990; Li et al., 2001; Quinta-Ferreira et al., 2004; Huang et al., 2008; Ketterman and Li, 2008; Liu et al., 2008; Hershfinkel et al., 2009; Sindreu et al., 2011; Khan et al., 2014; Vergnano et al., 2014; Woodier et al., 2015; Ha et al., 2018; Levaot and Hershfinkel, 2018; Sanford et al., 2019; Morabito et al., 2022). Ca^2+^ dependent Zn^2+^ release is well-documented in several studies. It has been shown that labile Zn^2+^ is abundant in a subset of glutamatergic neurons, specifically localized in synaptic vesicles and, like conventional neurotransmitters, Zn^2+^ is released in a depolarization and Ca^2+^ dependent manner (Frederickson et al., 2000; Li et al., 2001; Khan et al., 2014). Additionally, it has been shown that stimulation with KCl or glutamate in dissociated hippocampal neurons induces a rise in [Zn^2+^]i that is downstream from Ca^2+^ signaling and that the source of Zn^2+^ release is from intracellular stores (Kiedrowski, 2012; Sanford et al., 2019; Sanford and Palmer, 2020). The other way of interaction also exists, whereby Zn^2+^ modulates Ca^2+^ dynamic and signaling in excitable cells. It has been shown that intracellular Zn^2+^ has a higher affinity to bind to the cardiac ryanodine receptor (RYR) than Ca^2+^ and that a millimolar rise in intracellular levels of free Zn^2+^ inhibits the channel opening, thereby regulating Ca^2+^ release (Woodier et al., 2015). In addition, Zn^2+^ dependent Ca^2+^ influx in cortical neurons has been shown to contribute to neuronal cell death during oxidative stress (Park et al., 2019).

Our study revealed a possible interaction between intracellular Ca^2+^ and Zn^2+^ signaling in response to EFS. EFS in the absence of extracellular Ca^2+^ evoked significantly higher maximum release of intracellular Zn^2+^, indicating that extracellular Ca^2+^ may interfere with intracellular release of Zn^2+^. It is therefore tempting to speculate that, similar to the Zn^2+^ modulation of the cardiac RYR, Ca^2+^ entry through voltage sensitive Ca^2+^ channels may be also modulated and/or inhibited by binding of intracellular Zn^2+^ and that in the absence of extracellular Ca^2+^, Zn^2+^ will not bind to these channels leading to higher levels of free Zn^2+^ (Sun et al., 2007; Woodier et al., 2015). This may also explain the reduced release of intracellular Ca^2+^ in response to EFS in the presence of extracellular Ca^2+^. Further investigations are required to decipher details of this interdependence relationship between Ca^2+^ and Zn^2+^ and to elucidate the downstream signaling and determine its consequence.

In this study, we normalized fluorescence intensity to baseline in order to quantify the relative changes in intracellular Ca^2+^ and Zn^2+^. Normalization to baseline assumes that the treatment does not affect baseline levels, which might not be always true. We therefore provided evidence of the effect of the various treatments on baseline values (Supplementary Figure 5). We show that cellular Fluo-4 AM fluorescence intensity of cells bathed in calcium-free Krebs medium exhibited no apparent difference from that of cells bathed in normal calcium medium indicating no effect on baseline level of Fluo-4 AM fluorescence (Supplementary Figure 5A). Manipulation of extracellular calcium would lead to changes in intracellular calcium set-point (al-Mohanna et al., 1992) however, the relatively short time of exposure to calcium free-medium might explain the lack of effect we have noticed. In addition, the possibility that these cultured cortical neurons might be more resistant to changes in extracellular calcium than others (within the time scale of the experiments), could not be excluded. Future research would clear this point. On the other hand, we have found a significant difference in the baseline Fluozin-1 AM intensity levels under the different extracellular calcium levels (Supplementary Figure 5B). As for TPEN treatment, we found no statistical difference in the Fluozin-1 AM intensity levels in cells treated with TPEN compared to untreated cells (Supplementary Figure 5C).

In summary, we showed that modifying membrane potential of cortical neurons by EFS resulted in hyperpolarization that is concurrently accompanied by release of Ca^2+^ and Zn^2+^ from intracellular sources. Our study highlights an association between EFS-induced modification of membrane excitability and [Ca^2+^]_i_ and [Zn^2+^]_i_ signaling and supports the use of EFS as a tool to interrogate the dynamics of intracellular ions homeostasis.

## Data availability statement

The raw data supporting the conclusions of this article will be made available by the authors, without undue reservation.

## Ethics statement

The animal study was reviewed and approved by the Animal Care and Use Committee of the King Faisal Specialist Hospital and Research Centre.

## Author contributions

AA and FAM: conception and design of the study, data analysis and interpretation, and manuscript writing. AA, SA, and FAM: collection and/or assembly of data. FAM: provision of study material and administrative and financial support. All authors contributed to final approval of the manuscript.

## Supplementary material

Supplementary Figure 1 Extended experiment duration for electric field stimulation (EFS) induced zinc (Zn^2+^) changes in regular Krebs, calcium (Ca^2+^) free Krebs and TPEN supplemented Krebs media. The graph shows average intensity changes of Zn^2+^ indicator Fluozin-1 AM in response to EFS in regular Krebs buffer (green), in Krebs buffer depleted from Ca^2+^ (blue) and in TPEN supplemented Krebs medium (orange) for an extended duration corresponding to 400 s. The EFS was applied at second 20 of the experiment for a duration of 20 s. Data shown represents the average of two or more independent experiments and is expressed as normalized fluorescence intensity ratio relative to the averaged three images obtained prior to EFS.

Supplementary Figure 2 Electric field stimulation (EFS) induced membrane potential changes in regular Krebs medium compared to calcium (Ca^2+^) free Krebs medium. The graph shows average intensity changes of the membrane potential dye DiBAC4(3) in response to EFS with ± 5 V in regular Krebs (orange) and calcium free Krebs (blue). The EFS was applied at second 20 of the experiment for a duration of 20 s. Data shown represent the average of two independent experiments and is expressed as normalized fluorescence intensity ratio relative to the averaged three images obtained prior to EFS.

Supplementary Figure 3 The effect of Diazoxide on intracellular calcium and zinc. **(A)** Sequential raw confocal images of Fluo-4 AM labeled cortical cells at the indicated time intervals corresponding to before, during and after exposure to Diazoxide (10 μM) in regular Krebs buffer. Diazoxide (10 μM) was added at second 20 of the time lapse recording experiment. Scale bar = 20 μm. **(B)** Sequential raw confocal images of Fluozin-1 AM labeled cortical cells at the indicated time intervals corresponding to before, during and after exposure to Diazoxide (10 μM) in regular Krebs buffer. Diazoxide (10 μM) was added at second 20 of the time lapse recording. Scale bar = 20 μm. **(C)** The graph shows average intensity changes of Ca^2+^ indicator Fluo-4 AM in response to exposure to Diazoxide (10 μM) in regular Krebs buffer (blue) and in Krebs buffer depleted from Ca^2+^ (red). Diazoxide (10 μM) was added at second 20, followed by the addition of Ca^2+^ Ionophore at second 200 to obtain maximum Ca^2+^ levels as a control, and finally EGTA to chelate Ca^2+^ at second 400. Data shown represents the average of one independent experiment and is expressed as normalized fluorescence intensity ratio relative to the averaged three images obtained prior to the addition of Diazoxide.

Supplementary Figure 4 Extended experiment duration for electric field stimulation (EFS) induced calcium (Ca^2+^) changes in Ca^2+^ free Krebs medium. The graph shows average intensity changes of Ca^2+^ indicator Fluo-4 AM in response to EFS in in Krebs buffer depleted from Ca^2+^ for an extended duration corresponding to 400 s. The EFS was applied at second 20 of the experiment for a duration of 20 s. Data shown represents the average of three independent experiments and is expressed as normalized fluorescence intensity ratio relative to the averaged three images obtained prior to EFS.

Supplementary Figure 5 Effects of calcium free Krebs-HEPES medium and TPEN on baseline fluorescence intensity levels. **(A)** Mean fluorescence intensity of cells loaded with Fluo-4 AM bathed in Krebs-HEPES buffer containing 1.2 mM Ca^2+^ (Fluo4 in Norm Ca^2+^) and Krebs-HEPES buffer without Ca^2+^ and in the presence of 1 mM EGTA (Fluo4 No Ca^2+^). Data presented as mean ± SEM, *n* = 27 and 28 cells obtained from three independent experiments, respectively. **(B)** Mean fluorescence intensity of cells loaded with Fluozin-1 AM bathed in Krebs-HEPES buffer containing 1.2 mM Ca^2+^ (FZ Norm Ca^2+^) and Krebs-HEPES buffer without Ca^2+^ and in the presence of 1mM EGTA (FZ No Ca^2+^). Data presented as mean ± SEM, *n* = 41 and 65 cells obtained from three independent experiments, respectively. **(C)** Mean fluorescence intensity of cells loaded with Fluozin-1 AM bathed in Krebs-HEPES buffer containing 1.2 mM Ca^2+^ treated with 50 μM TPEN (With TPEN) compared to untreated cells (carrier only), (Without TPEN). Data are presented as mean ± SEM, *n* = 8 and 41 cells obtained from two and three independent experiments, respectively.
