# Supplementary figures and images for: The interplay of intracellular calcium and zinc ions in response to electric field stimulation in primary rat cortical neurons in vitro
Source: Front Cell Neurosci. 2023 Apr 27;17:1118335. doi: 10.3389/fncel.2023.1118335 (PMC10174245; doi:10.3389/fncel.2023.1118335)

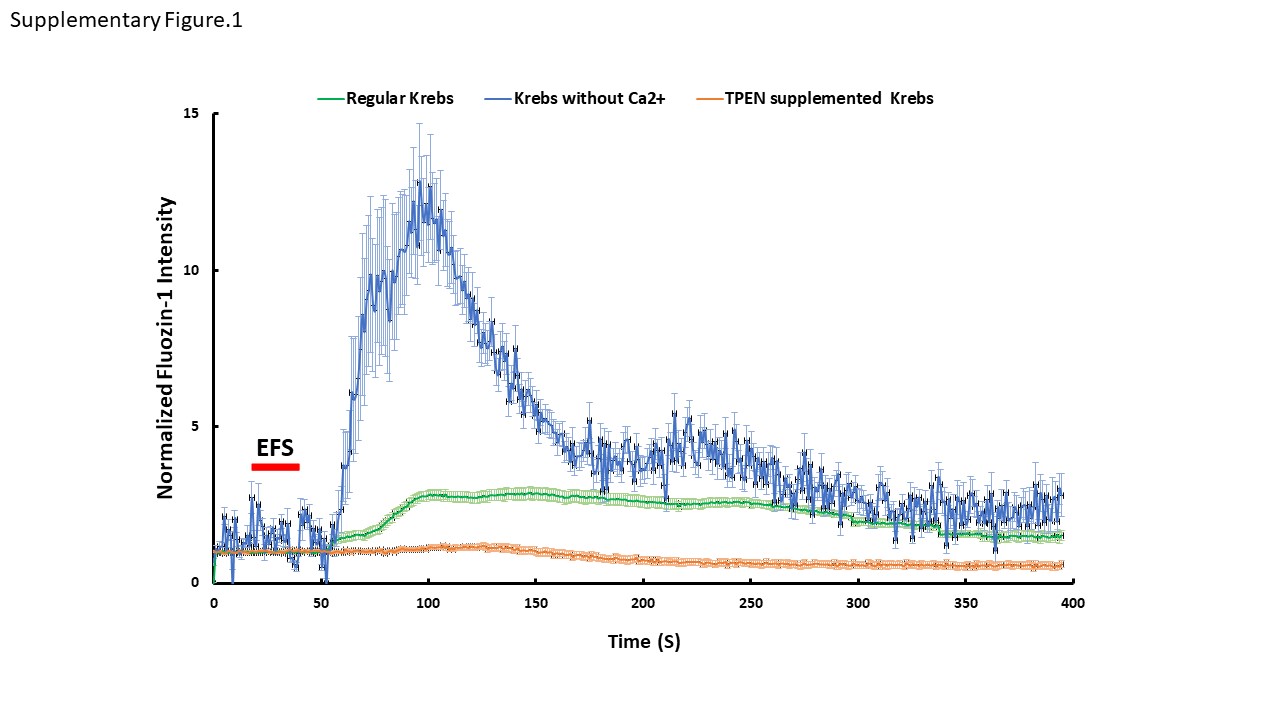

Supplement: Supplementary Figure 1 — Extended experiment duration for electric field stimulation (EFS) induced zinc (Zn2+) changes in regular Krebs, calcium (Ca2+) free Krebs and TPEN supplemented Krebs media. The graph shows average intensity changes of Zn2+ indicator Fluozin-1 AM in response to EFS in regular Krebs buffer (green), in Krebs buffer depleted from Ca2+ (blue) and in TPEN supplemented Krebs medium (orange) for an extended duration corresponding to 400 s. The EFS was applied at second 20 of the experiment for a duration of 20 s. Data shown represents the average of two or more independent experiments and is expressed as normalized fluorescence intensity ratio relative to the averaged three images obtained prior to EFS. [file Image_1.JPEG]

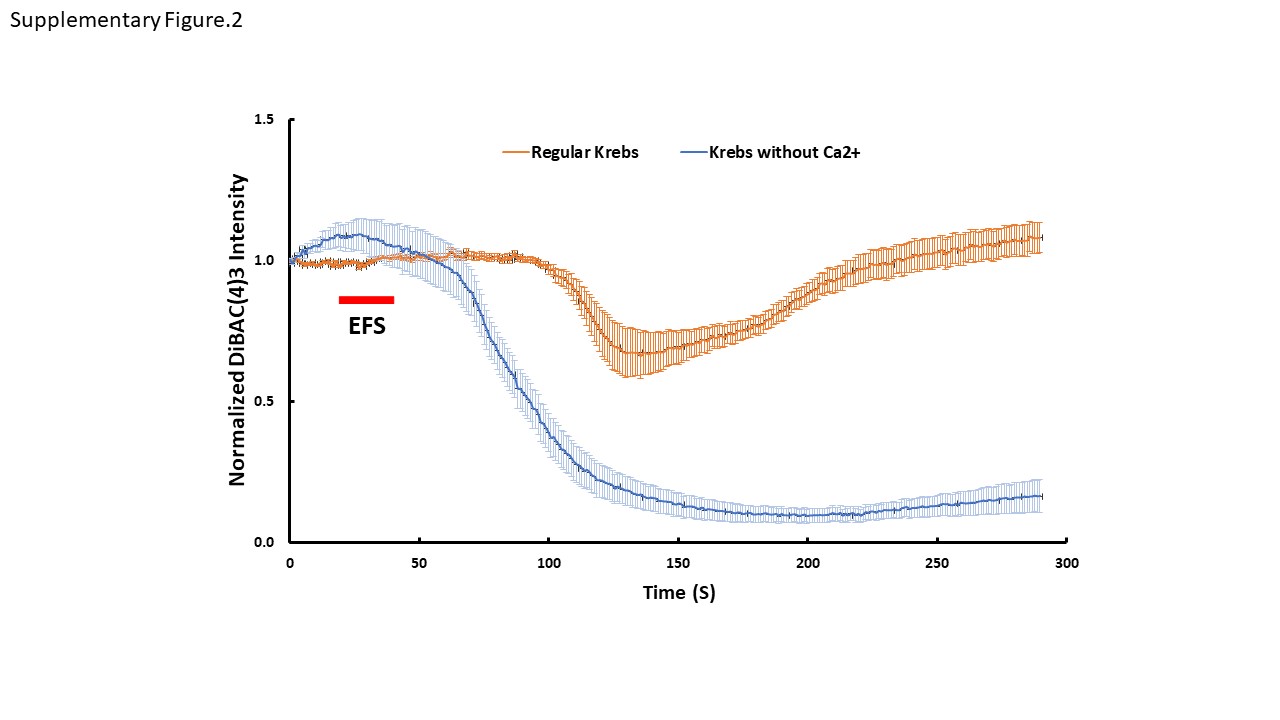

Supplement: Supplementary Figure 2 — Electric field stimulation (EFS) induced membrane potential changes in regular Krebs medium compared to calcium (Ca2+) free Krebs medium. The graph shows average intensity changes of the membrane potential dye DiBAC4(3) in response to EFS with ± 5 V in regular Krebs (orange) and calcium free Krebs (blue). The EFS was applied at second 20 of the experiment for a duration of 20 s. Data shown represent the average of two independent experiments and is expressed as normalized fluorescence intensity ratio relative to the averaged three images obtained prior to EFS. [file Image_2.JPEG]

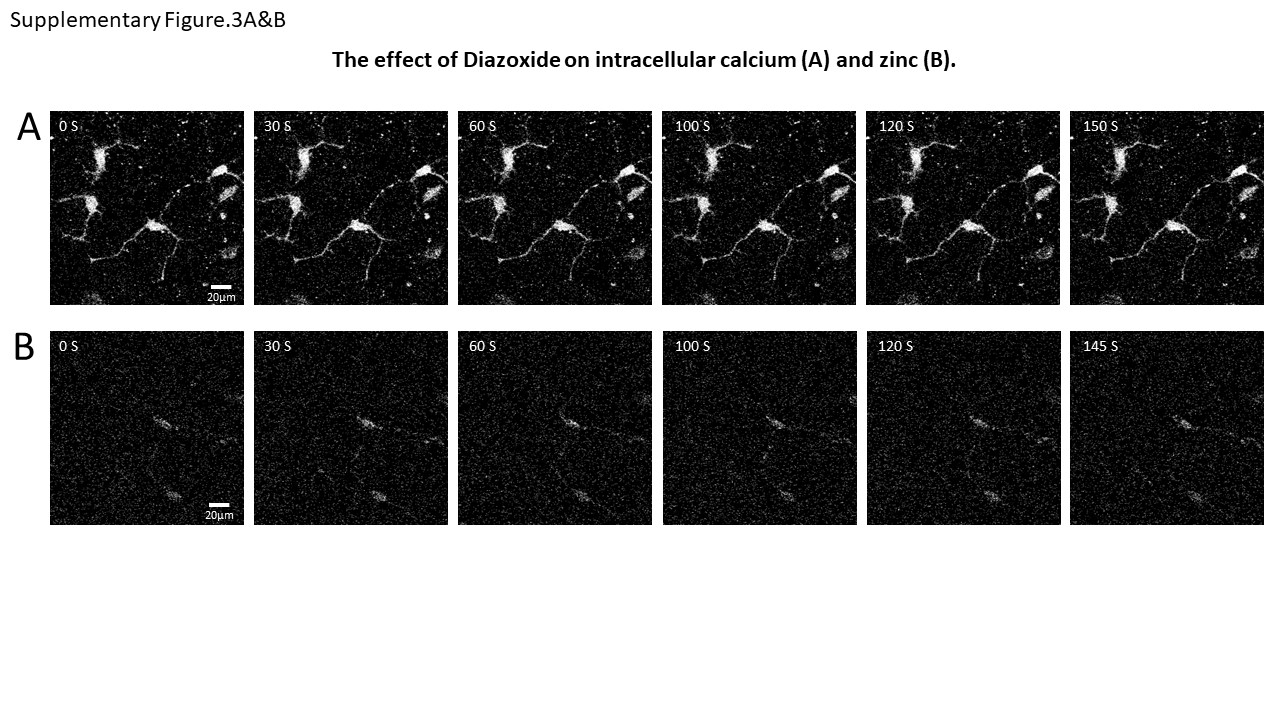

Supplement: Supplementary Figure 3 — The effect of Diazoxide on intracellular calcium and zinc. (A) Sequential raw confocal images of Fluo-4 AM labeled cortical cells at the indicated time intervals corresponding to before, during and after exposure to Diazoxide (10 μM) in regular Krebs buffer. Diazoxide (10 μM) was added at second 20 of the time lapse recording experiment. Scale bar = 20 μm. (B) Sequential raw confocal images of Fluozin-1 AM labeled cortical cells at the indicated time intervals corresponding to before, during and after exposure to Diazoxide (10 μM) in regular Krebs buffer. Diazoxide (10 μM) was added at second 20 of the time lapse recording. Scale bar = 20 μm. (C) The graph shows average intensity changes of Ca2+ indicator Fluo-4 AM in response to exposure to Diazoxide (10 μM) in regular Krebs buffer (blue) and in Krebs buffer depleted from Ca2+ (red). Diazoxide (10 μM) was added at second 20, followed by the addition of Ca2+ Ionophore at second 200 to obtain maximum Ca2+ levels as a control, and finally EGTA to chelate Ca2+ at second 400. Data shown represents the average of one independent experiment and is expressed as normalized fluorescence intensity ratio relative to the averaged three images obtained prior to the addition of Diazoxide. [file Image_3.JPEG]

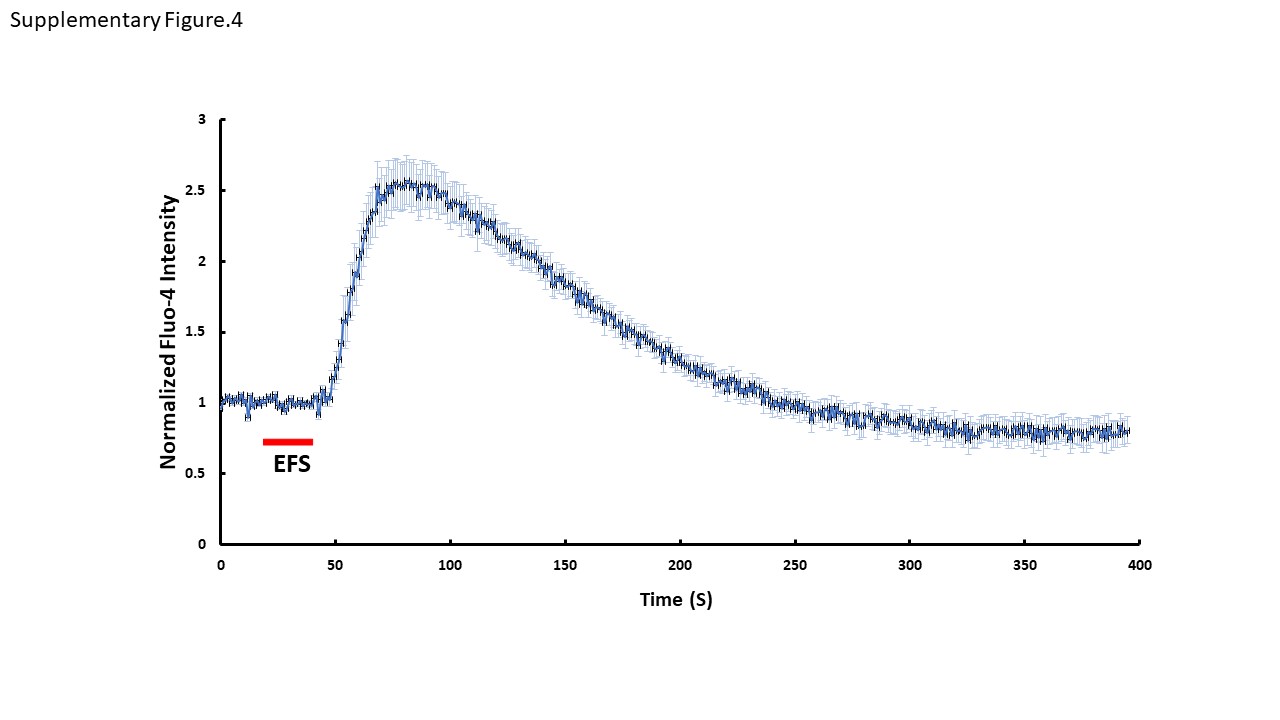

Supplement: Supplementary Figure 4 — Extended experiment duration for electric field stimulation (EFS) induced calcium (Ca2+) changes in Ca2+ free Krebs medium. The graph shows average intensity changes of Ca2+ indicator Fluo-4 AM in response to EFS in in Krebs buffer depleted from Ca2+ for an extended duration corresponding to 400 s. The EFS was applied at second 20 of the experiment for a duration of 20 s. Data shown represents the average of three independent experiments and is expressed as normalized fluorescence intensity ratio relative to the averaged three images obtained prior to EFS. [file Image_5.JPEG]

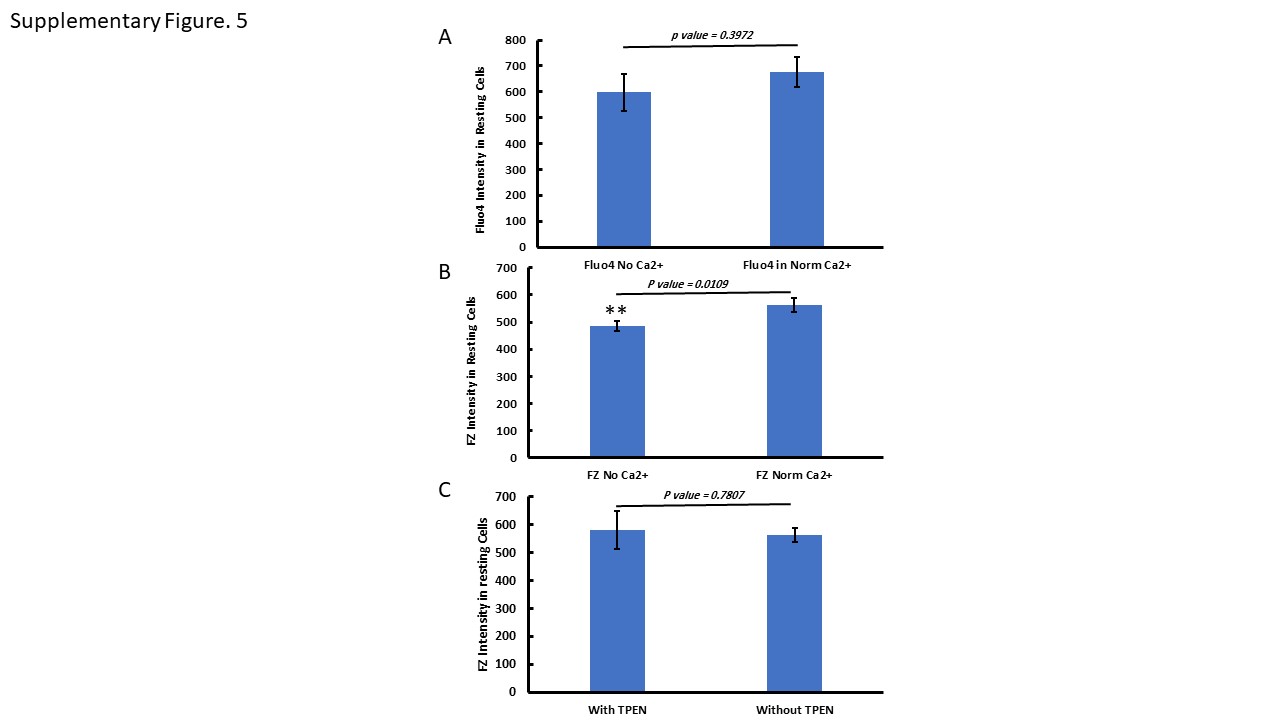

Supplement: Supplementary Figure 5 — Effects of calcium free Krebs-HEPES medium and TPEN on baseline fluorescence intensity levels. (A) Mean fluorescence intensity of cells loaded with Fluo-4 AM bathed in Krebs-HEPES buffer containing 1.2 mM Ca2+ (Fluo4 in Norm Ca2+) and Krebs-HEPES buffer without Ca2+ and in the presence of 1 mM EGTA (Fluo4 No Ca2+). Data presented as mean ± SEM, n = 27 and 28 cells obtained from three independent experiments, respectively. (B) Mean fluorescence intensity of cells loaded with Fluozin-1 AM bathed in Krebs-HEPES buffer containing 1.2 mM Ca2+ (FZ Norm Ca2+) and Krebs-HEPES buffer without Ca2+ and in the presence of 1mM EGTA (FZ No Ca2+). Data presented as mean ± SEM, n = 41 and 65 cells obtained from three independent experiments, respectively. (C) Mean fluorescence intensity of cells loaded with Fluozin-1 AM bathed in Krebs-HEPES buffer containing 1.2 mM Ca2+ treated with 50 μM TPEN (With TPEN) compared to untreated cells (carrier only), (Without TPEN). Data are presented as mean ± SEM, n = 8 and 41 cells obtained from two and three independent experiments, respectively. [file Image_6.JPEG]

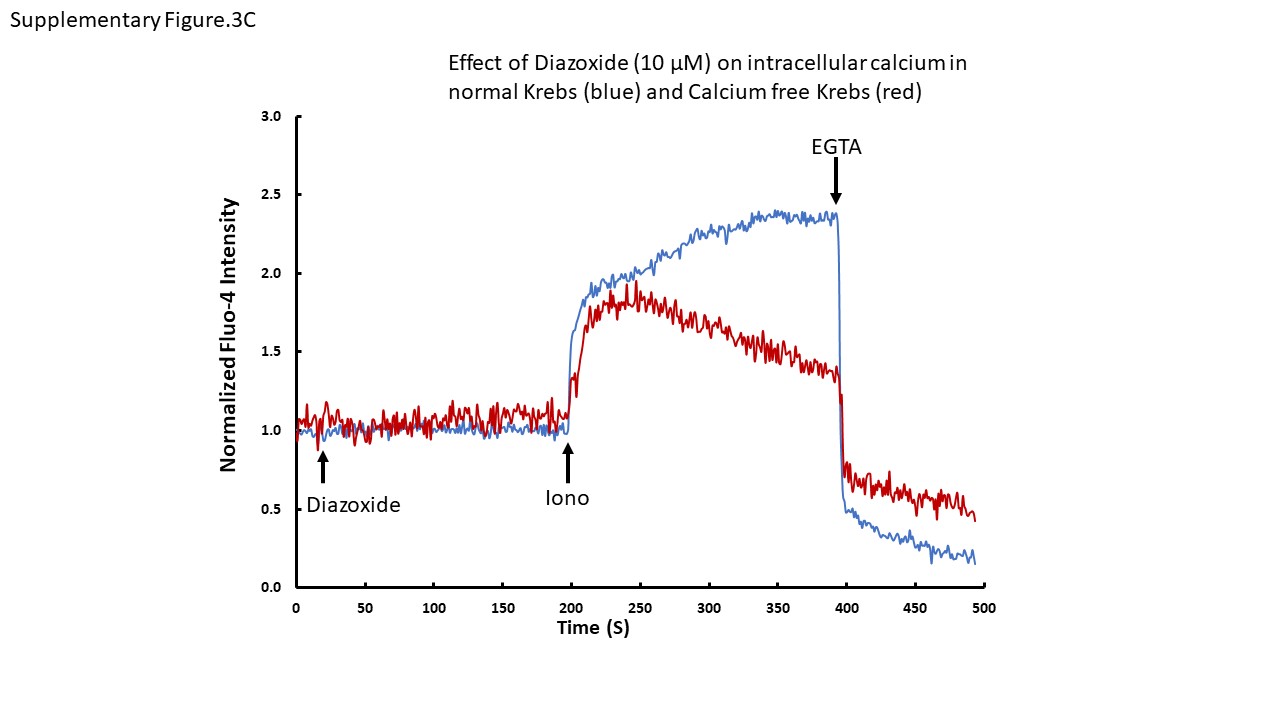

Supplement: Supplementary file 6 [file Image_4.JPEG]
